# P-225. Effect of isolating *Clostridioides difficile* asymptomatic carriers on the incidence of healthcare-associated *C. difficile* infections: a systematic review and meta-analysis

**DOI:** 10.1093/ofid/ofae631.429

**Published:** 2025-01-29

**Authors:** Veronica Zanichelli, Jacqueline Wong, Zahra Sohani, Nick Daneman, Charles Frenette, Todd C Lee, Dominik Mertz, Emily McDonald, Kevin Brown, Jennifer M Grant, Louis-Patrick Haraoui, Jennie Johnstone, Kevin Katz, Robyn S Lee, Jerome A Leis, Beate Sander, Titus Wong, Vivian Loo, Yves Longtin

**Affiliations:** McGill University, MONTREAL, Quebec, Canada; McMaster University, Hamilton, Ontario, Canada; Division of Infectious Diseases, Department of Medicine, McGill University Health Centre, Montreal, Quebec, Canada; Sunnybrook Health Sciences Centre, University of Toronto, Toronto, Ontario, Canada; McGill University Health Centre, Montreal, Quebec, Canada, Montreal, QC, Canada; McGill University, MONTREAL, Quebec, Canada; McMaster University, Hamilton, Ontario, Canada, Hamilton, ON, Canada; McGill University Health Centre, Montreal, Quebec, Canada; University of Toronto, Toronto, Ontario, Canada; University of British Columbia Faculty of medicine, division of pathology and laboratory medicine, Vancouver, British Columbia, Canada; Sherbrooke University Faculty of Medicine and Hopital Charles-Lemoyne, Sherbrooke, Quebec, Canada; University of Toronto, Toronto, Ontario, Canada; North York General Hospital, Toronto, Ontario, Canada; University of Toronto Dalla Lana School of Public Health, Toronto, Ontario, Canada; Sunnybrook Health Sciences Centre, Toronto, Ontario, Canada; University Health Network, Toronto, Ontario, Canada; Vancouver Coastal Health Authority, Vancouver, British Columbia, Canada; McGill University, MONTREAL, Quebec, Canada; Jewish General Hospital, Montreal, Montreal, QC, Canada

## Abstract

**Background:**

Asymptomatic carriers are a potential source of healthcare-associated *Clostridioides difficile* infection (HA-CDI). Our objective was to investigate the impact of interventions to detect and isolate asymptomatic carriers during their hospital stay on the incidence of HA-CDI.

**Figure 1:**
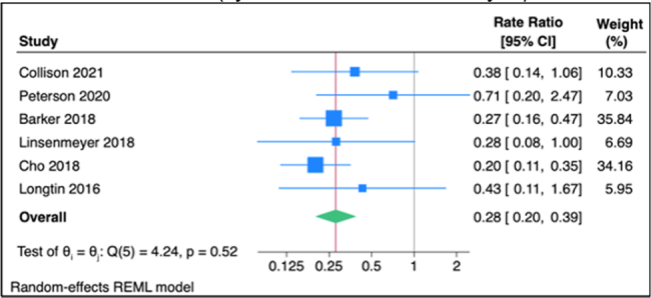
Forest plot of the association between screening and isolating C. difficile carriers on admission and HA-CDI (by random-effects meta-analysis)

**Methods:**

We performed a systematic review and searched the research literature up to March 2023 for interventional studies where asymptomatic adult patients were screened for *C. difficile* colonization and were isolated if positive. Only studies that allocated interventions to clusters of individuals and measured outcomes at the population level were eligible. HA-CDI incidence rate ratios were pooled through random effects meta-analysis. Risk of bias was assessed using the Cochrane’s tool for non-randomized studies of interventions.

**Figure 2:**
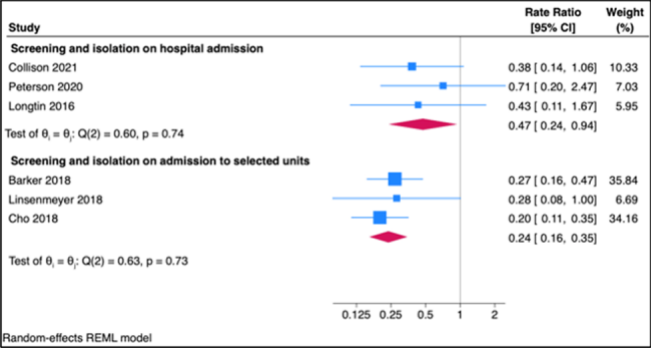
Forest plot of the association between screening and isolating C. difficile carriers on admission to hospital or to selected units and HA-CDI (by random-effects meta-analysis)

**Results:**

5,681 unique references were screened of which six were eligible. All studies were before-after quasi-experimental interventional studies (2/6 included a control group and 4/6 compared incidence rates during the intervention to before) from high-income countries published between 2016 and 2020 that assessed the effects of hospital-wide or unit-wide screening. Two studies only screened patients at high risk of CDI (e.g. hematologic patients receiving chemotherapy). Four studies were considered to have a serious risk of bias. Among the six included studies, 75,870 patients were screened and 4,104 patients were identified as carriers (the proportion of carriers ranged from 3.1 to 15%). Using a random-effects inverse-variance meta-analysis, screening and isolation of carriers was associated with a significant reduction in the incidence of HA-CDI (N=6, pooled rate ratio 0.28, 95% CI 0.20-0.39). Subgroup analyses yielded similar point estimates for reduction in HA-CDI rates both when screening was done on hospital admission (N=3, RR 0.47, 95% CI 0.24 to 0.94) and on admission to specific hospital units (N=3, RR 0.24, 95% CI 0.16 to 0.34).

**Conclusion:**

Based on this systematic review and meta-analysis, screening and isolating *C. difficile* carriers is associated with a decreased incidence of HA-CDI in adult patients. Due to methodological limitations, robust randomized controlled trials are needed to confirm this finding.

**Disclosures:**

**Dominik Mertz, MD, MSc**, KCI Inc. USA: Grant/Research Support

